# Self-Reported COVID-19 Vaccine and Booster Acceptance and Hesitancy Among Autistic Adults in Pennsylvania: Cross-Sectional Analysis of Survey Data

**DOI:** 10.2196/51054

**Published:** 2024-08-28

**Authors:** Lindsay Shea, Dylan Cooper, Jonas Ventimiglia, Shelby Frisbie, Conner Carlton, Wei Song, Mark Salzer, Brian Lee, Emily Hotez, David J Vanness

**Affiliations:** 1 AJ Drexel Autism Institute Drexel University Philadelphia, PA United States; 2 College of Public Health Temple University Philadelphia, PA United States; 3 Dornsife School of Public Health Drexel University Philadelphia, PA United States; 4 David Geffen School of Medicine University of California Los Angeles Los Angeles, CA United States; 5 College of Health and Human Development Pennsylvania State University University Park, PA United States

**Keywords:** autism, COVID-19, vaccination, public health, autism spectrum disorder, autistic, vaccine, vaccines, acceptance, hesitancy, immunize, immunization, immunizations, attitude, attitudes, opinion, perception, perceptions, perspective, perspectives, neurodevelopmental, infectious, respiratory, survey, surveys

## Abstract

**Background:**

The autistic population is rapidly increasing; meanwhile, autistic adults face disproportionate risks for adverse COVID-19 outcomes. Limited research indicates that autistic individuals have been accepting of initial vaccination, but research has yet to document this population’s perceptions and acceptance of COVID-19 boosters.

**Objective:**

This study aims to identify person-level and community characteristics associated with COVID-19 vaccination and booster acceptance among autistic adults, along with self-reported reasons for their stated preferences. Understanding this information is crucial in supporting this vulnerable population given evolving booster guidelines and the ending of the public health emergency for the COVID-19 pandemic.

**Methods:**

Data are from a survey conducted in Pennsylvania from April 11 to September 12, 2022. Demographic characteristics, COVID-19 experiences, and COVID-19 vaccine decisions were compared across vaccination status groups. Chi-square analyses and 1-way ANOVA were conducted to test for significant differences. Vaccination reasons were ranked by frequency; co-occurrence was identified using phi coefficient correlation plots.

**Results:**

Most autistic adults (193/266, 72.6%) intended to receive or received the vaccine and booster, 15% (40/266) did not receive or intend to receive any vaccine, and 12.4% (33/266) received or intended to receive the initial dose but were hesitant to accept booster doses. Reasons for vaccine acceptance or hesitancy varied by demographic factors and COVID-19 experiences. The most significant were previously contracting COVID-19, desire to access information about COVID-19, and discomfort with others not wearing a mask (all *P*=.001). County-level factors, including population density (*P*=.02) and percentage of the county that voted for President Biden (*P*=.001) were also significantly associated with differing vaccination acceptance levels. Reasons for accepting the initial COVID-19 vaccine differed among those who were or were not hesitant to accept a booster. Those who accepted a booster were more likely to endorse protecting others and trusting the vaccine as the basis for their acceptance, whereas those who were hesitant about the booster indicated that their initial vaccine acceptance came from encouragement from someone they trusted. Among the minority of those hesitant to any vaccination, believing that the vaccine was unsafe and would make them feel unwell were the most often reported reasons.

**Conclusions:**

Intention to receive or receiving the COVID-19 vaccination and booster was higher among autistic adults than the population that received vaccines in Pennsylvania. Autistic individuals who accepted vaccines prioritized protecting others, while autistic individuals who were vaccine hesitant had safety concerns about vaccines. These findings inform public health opportunities and strategies to further increase vaccination and booster rates among generally accepting autistic adults, to better support the already strained autism services and support system landscape. Vaccination uptake could be improved by leveraging passive information diffusion to combat vaccination misinformation among those not actively seeking COVID-19 information to better alleviate safety concerns.

## Introduction

Equity-centered approaches are crucial to support vulnerable populations throughout the COVID-19 pandemic as vaccination and booster recommendations continue evolving. Evidence-based public health messaging promoting vaccine literacy and acceptance among high-risk groups is urgently needed, especially in the wake of newly released and continued deployment of booster vaccines developed to combat increasing variants of the COVID-19 virus [1]. Understanding the factors influencing decisions to get vaccinated and boosted is critical to developing effective content and delivery modalities for public health messaging. Autistic adults represent a growing, high-risk group. Now, 2.8% of the US population meets diagnostic criteria for autism [2], and recent research has documented their increased risk for complications and adverse outcomes from COVID-19 [3,4]. This aligns with research among the wider adult disability community that found nearly 1.2 times greater risk of contracting COVID-19 compared to individuals without disabilities [5].

There is a complicated history between autism and vaccines. However, this is rooted in debunked theories about early childhood vaccinations and not adult vaccinations [6], leaving the impact of COVID-19 vaccination decision-making on autistic adults a critical area of inquiry to best support this high-risk population. Two existing studies have found that autistic adults had similar levels of initial COVID-19 vaccine acceptance compared to peers without autism [7,8]. A desire to protect others, fear of the COVID-19 pandemic, and overall trust in the vaccine were commonly endorsed by vaccine-accepting autistic adults. Among vaccine-hesitant autistic adults, vaccine safety and side effects were listed as the greatest worries [7,8], outweighing fears of getting seriously ill from COVID-19 [7]. Parent and caregiver perspectives were also found to be an influential factor that could sway both acceptance and hesitance [8].

Political party affiliation has also been found to be a strong predictor of vaccination preferences [9-11], with data indicating that the COVID-19 cases and death rates are higher in counties with Republican voter majorities [12], and that this gap has increased since the release of COVID-19 vaccines [13]. Evidence suggests that these trends may similarly impact autistic adults, as a significantly greater proportion of vaccine-accepting autistic adults reported living in counties that President Biden won in the 2020 election [7]. This may be due to information streams as conservative outlets like Fox News disseminated language that downplayed the severity of the virus and suggested that the COVID-19 pandemic was a ploy to harm President Trump’s 2020 reelection campaign efforts [14].

Given the dynamic, multifaceted nature of the pandemic—including relaxation or discontinuation of incentives and mandates for COVID-19 vaccinations [15]; evolving, often partisan perspectives on COVID-19 mitigation measures; rampant COVID-19 misinformation [16]; altered expectations for vaccine efficacy given the emergence of new variants, waning immunity, and breakthrough infections [17]; and mixed messages about relevance and urgency in the light of recent public health actions [18]—additional data are needed on the impact of the effectiveness of vaccine-related public health messaging as it relates to promoting booster acceptancy. Some emerging research outside of the United States has indicated that respondents are generally accepting to receiving additional COVID-19 boosters [19,20]. Nevertheless, the transferability of these results to the United States is unclear given how uniquely politically fraught COVID-19 mitigation and vaccination efforts are in the country [9-11]. This is especially true because booster rates continue to remain low in the general population [21], with a study finding similar trends among Americans with disabilities [22], and research on booster acceptance and hesitancy among autistic adults remains scant despite their disproportionate risk for adverse outcomes. One existing global study that includes some autistic adult participants [8], however, found that less than half of the autistic respondents received a booster, despite high rates of initial vaccination. It could be that the need for continued COVID-19 vaccines is uniquely impacting autistic individuals, necessitating further inquiry.

Public health communication has consistently identified that vaccine misinformation is especially detrimental for individuals with disabilities, including diagnoses like autism that include social and communication differences or challenges [23]. A recent qualitative study found that misinformation is directly contributing to vaccine hesitancy in neurodivergent communities [24]. Addressing this is critical as vaccination against COVID-19 remains one of the strongest repellants of adverse outcomes including hospitalization or death [25-27]. Public health messaging that responds to and incorporates evidence-based findings will be best equipped to improve booster acceptance, particularly across vulnerable populations like autistic adults. Therefore, this study sought to answer the following research questions: (1) What demographic, clinical, and community characteristics are associated with COVID-19 vaccination and booster uptake among autistic adults? and (2) What are the self-reported reasons for vaccination and booster acceptance or hesitancy? Findings to these questions will help illuminate barriers and facilitators of vaccination and support evidence-driven strategies for optimal public health messaging.

## Methods

### Sample and Data

Data were obtained from COVID-19–specific questions included in a follow-up survey of the Pennsylvania Autism Needs Assessment (PANA) [28]. Participants originally participated in the 2018 PANA, which collected data from autistic adults (self-report PANA) and caregivers (caregiver PANA) across life domains, including service needs, barriers, community participation, education experiences, and clinical and demographic information. The 2018 PANA recruited autistic adults and caregivers in 2 ways. First, autistic Pennsylvania residents were identified through Medicaid claims (ie, 1 inpatient or 2 outpatient Medicaid service or encounter claims that included an autism diagnosis, *International Classification of Diseases [ICD]-9* 299.XX or *ICD-10* F84.X) and invited to participate via a postal mail letter and survey link. Of note, autism spectrum disorder diagnoses obtained from claims data yield high sensitivity and specificity [29]. Second, respondents were recruited through Pennsylvania autism advocacy organizations. All participants were asked if they would participate in follow-up research.

All autistic adults from the 2018 self-report PANA who agreed to future contact (N=412) were invited to complete an additional survey with COVID-19 questions. Data for this cross-sectional analysis came from a follow-up survey conducted between April 2022 and September 2022 (n=266). Data from COVID-19 questions in a survey collected between March 2021 and September 2021 were included, as it provided initial data points from that period about vaccination perspectives. An additional group of PANA respondents who were caregivers of autistic adults were also recruited. Caregivers were asked to connect the research team to their adult child or dependent, who was invited to participate. Although caregivers could provide support for survey completion (eg, recording responses), autistic adults were required to self-report responses and to indicate if and how they were helped with the survey. The response rate among those who completed the self-report PANA was 31.1% (128/412). All individuals with complete data for questions regarding the COVID-19 pandemic (described below) were included in the analyses. To best protect the privacy of our autistic respondents, the data sets generated and analyzed during this study are only available from the corresponding author upon reasonable request.

### Ethical Considerations

This study was approved by the Drexel University institutional review board under protocol 2001007579. In order to participate in this study, all respondents were required to first document their consent for the study team after reviewing institutional review board–approved consent language, which included a description that all identifying information would remain completely confidential and that data collected may be deidentified and included in publications. If a participant needed support answering survey questions, a member of the study team was available to read questions and record responses. All participants were compensated with a US $40 Amazon gift card.

### Measures of Variables

Survey questions asked about COVID-19 experiences, vaccination, and booster status (received, scheduled or in the process of scheduling, or do not intend to receive), sources of information, and perspectives. COVID-19 questions were drawn from existing surveys and adapted through research team consensus [7] to ensure maximum accessibility and applicability for autistic respondents. Items assessing changes in daily living came from the Coronavirus Impact Scale [30]. Reasons for vaccination and booster uptake were drawn from a Kaiser Family Foundation (KFF) survey [31], while reasons for opposing vaccination used a questionnaire from Guidry et al [32]. One item on loneliness was from the COVID-19 Community Response Survey Guidance from Johns Hopkins University [33]. Questions related to previous COVID-19 diagnoses, comfort with masking, information-seeking habits and sources, and community participation impacts were created by the research team. For 60 respondents, their responses to their initial vaccination doses and their reasons for receiving the vaccine were carried forward from previous data collection in 2021 to reduce respondent burden and the potential for recall bias. These respondents were only asked about their booster vaccination status and reasons.

### Data Analysis Procedure

A total of 3 groups were defined from responses to questions about COVID-19 vaccination and booster status from data collected in 2022. *Vaccine and booster accepters* were those who received a COVID-19 vaccine, had an appointment scheduled to do so, or were in the process of seeking an appointment for the initial series and first booster vaccine. *Vaccine accepter and booster hesitant* individuals received an initial COVID-19 vaccine but opted against a booster and had no plans to obtain one. The *vaccine hesitant* group had not received any COVID-19 vaccine and had no intentions to get one.

Self-reported, person-level demographic characteristics and COVID-19 experiences and preferences were compared across groups. County-level 2020 US Census population and county-level certified percentage of the 2020 US presidential election votes won by President Biden in Pennsylvania were also included, as measures of population characteristics relevant to vaccine acceptance [34]. Tests of differences were conducted using chi-square analyses and 1-way ANOVA with significance defined at .05. All analyses were completed using R (version 4.2.2; R Core Team). Reasons for COVID-19 vaccination acceptance or hesitancy were ranked by frequency and co-occurrence was investigated with phi coefficient correlation plots. The latter was used given the use of binary categorical variables in this study [35], as opposed to Pearson correlation, which is used for continuous variables, or Spearman and Kendall coefficients, which result in positive and negative values and make interpretation challenging.

## Results

Overall, 72.6% (193/266) of survey respondents reported receiving or intending to receive both a COVID-19 vaccine and a COVID-19 booster vaccine (Table 1). Of the subsample of autistic individuals who were accepting of initial vaccination (n=226), fewer (n=33, 14.6%) autistic individuals who received an initial COVID-19 vaccine had no intentions of getting a booster dose. A similar proportion (40/266, 15%) of the full sample of autistic individuals opted against any COVID-19 vaccines.

We identified significant (*P=*.03) gender differences by vaccine status, such that the *vaccine hesitant* group respondents were more likely to be women (14/39, 36%). A higher proportion of men were categorized as *vaccine accepter, booster hesitant* (31/33, 94%) or *vaccine and booster accepters* (138/191, 72.3%). Insurance type and status were associated with overall vaccine and booster acceptance. Private insurance was overrepresented among those who were vaccine and booster accepters; public insurance (Medicaid) was overrepresented among vaccine hesitant respondents; and having a combination of public and private insurance was overrepresented among those who were vaccine accepters but booster hesitant (*P*=.004).

A greater proportion (12/39, 31%) of the *vaccine hesitant* respondents reported minor disruptions in their routines, including going to work or school, self-care, or other ways time is spent during the COVID-19 pandemic, which more than doubled the proportion of either *vaccine accepter* group (*P*=.045). The *vaccine and booster accepter* group reported the highest proportion of major changes due to the COVID-19 pandemic (32/193, 16.6%) compared to less than 10% (3/33, 9.1% and 3/39, 7.7%) for each remaining group. The majority of all groups reported no changes to medical care access, although only *vaccine accepter, booster hesitant* respondents unanimously reported no disruptions, yielding significant differences across groups (*P=.*02).

Nearly all groups reported some or no discomfort with others not masking (compared to discomfort most of the time or always). Over 1 in 4 (51/191, 26.7%) of the *vaccine and booster accepter* group indicated they were uncomfortable with others not masking most or all of the time, compared to 9% (3/33) of the *vaccine accepter, booster hesitant* and 3% (1/38) of the *vaccine hesitant* group (*P=.*001).

The proportion of each group with a previous COVID-19 diagnosis increased with vaccine and booster hesitancy. Almost half (19/40, 48%) of those in the *vaccine hesitant* group contracted COVID-19 previously. A total of 1 in 3 (11/33, 33%) individuals in the *vaccine accepter, booster hesitant* group contracted COVID-19 previously, and 1 in 5 (39/193, 20.2%) individuals in the *vaccine and booster accepter* group contracted COVID-19 previously (*P=*.001).

The groups reported a wide range of COVID-19 news sources. A substantially higher proportion (15/40, 38%) of *vaccine hesitant* individuals did not try to access information about COVID-19 when compared to those who were *vaccine accepter, booster hesitant* (9/33, 27%) or *vaccine and booster accepters* (24/193, 12.4%; *P=*.001). Higher population density (*P*=.02) and the percentage of votes for President Biden in the 2020 US presidential election (*P=.*001) were associated with increased vaccination and booster acceptance.

**Figure 1:**
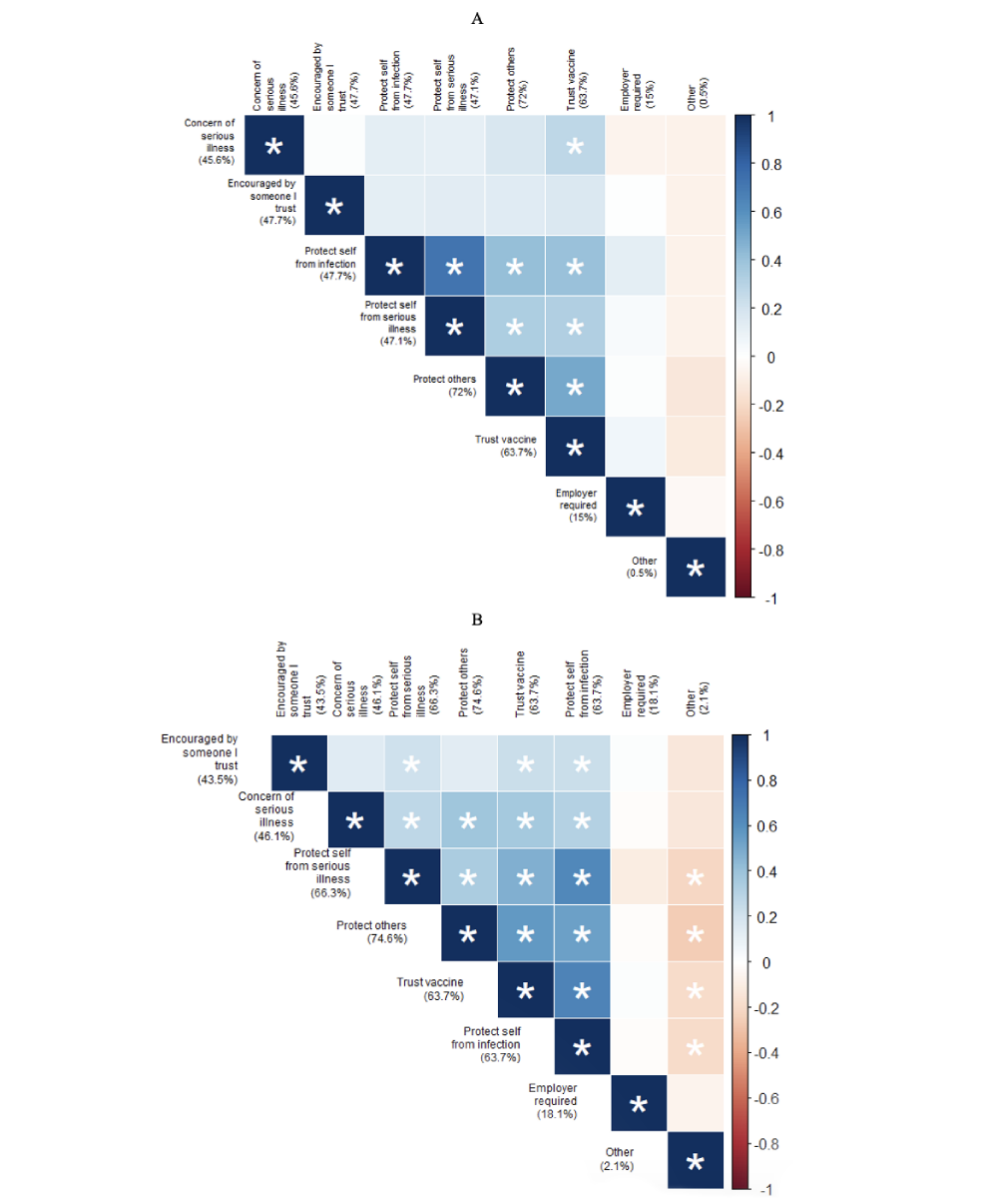
Correlation plots depicting associated and unassociated reasons for accepting both a COVID-19 vaccine and a COVID-19 booster among the subsample of autistic adults who were accepting of both in Pennsylvania during 2022. A: Vaccine and booster accepter reasons for getting the vaccine; B: Vaccine and booster accepter reasons for getting the booster. The color of each block represents the strength of the phi coefficient representing a correlation between the intersecting reasons. Phi coefficients with *P*<.05 are symbolized by an asterisk (*). Blue shades capture a tendency for the intersecting reasons to both be reported, contrary to red shades that visualize a tendency for only 1 of the reasons to be reported.

**Figure 2:**
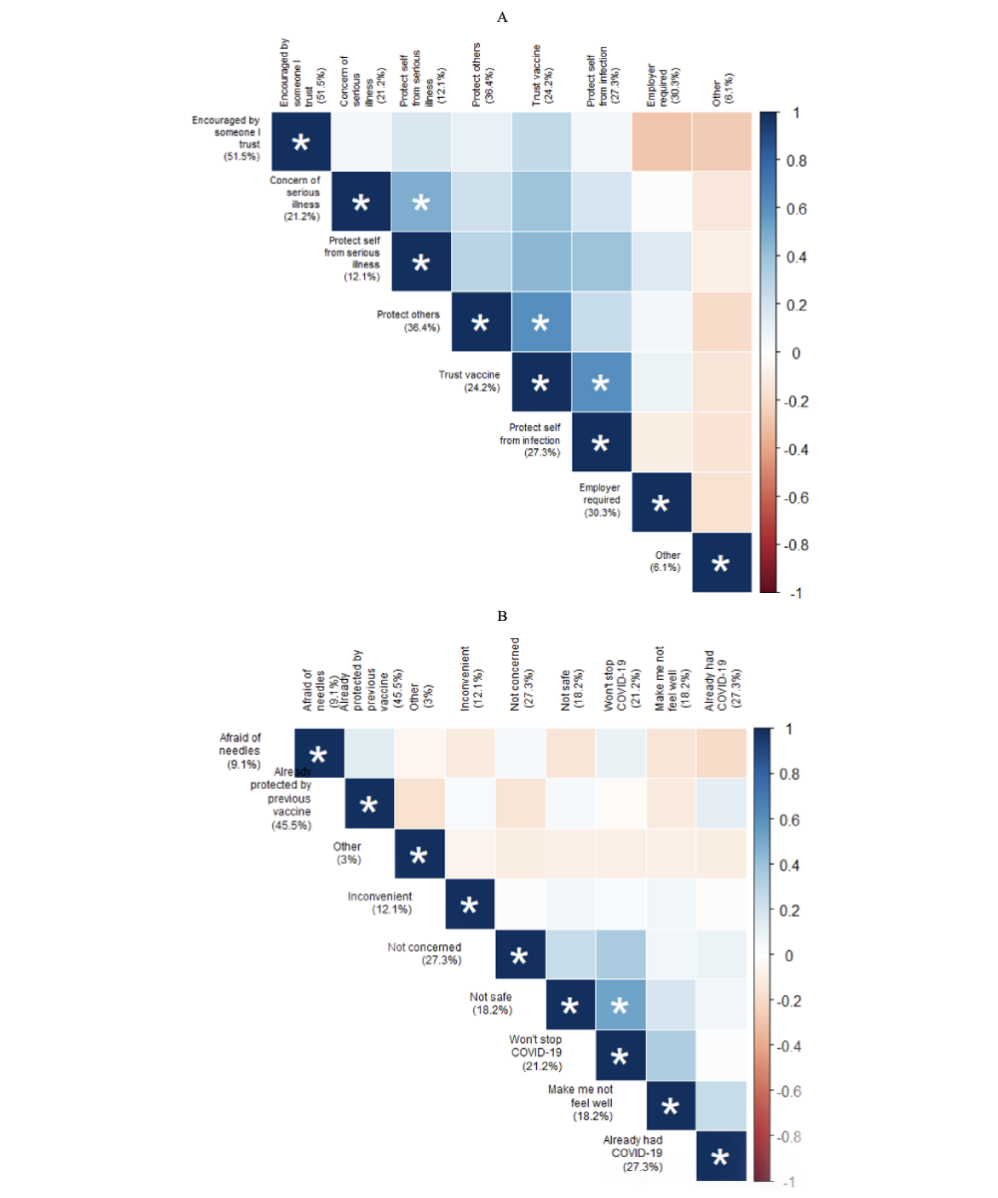
Correlation plots depicting associated and unassociated reasons for accepting a COVID-19 vaccine but expressing hesitancy to a COVID-19 booster among the subsample of autistic adults who were accepting a COVID-19 vaccine but hesitant toward a COVID-19 booster in Pennsylvania during 2022. A: Vaccine and booster accepter reasons for getting the booster; B: Vaccine accepter but booster hesitant reasons for getting the vaccine. The color of each block represents the strength of the phi coefficient representing a correlation between the intersecting reasons. Phi coefficients with *P*<.05 are symbolized by an asterisk (*). Blue shades capture a tendency for the intersecting reasons to both be reported, contrary to red shades that visualize a tendency for only 1 of the reasons to be reported.

**Figure 3:**
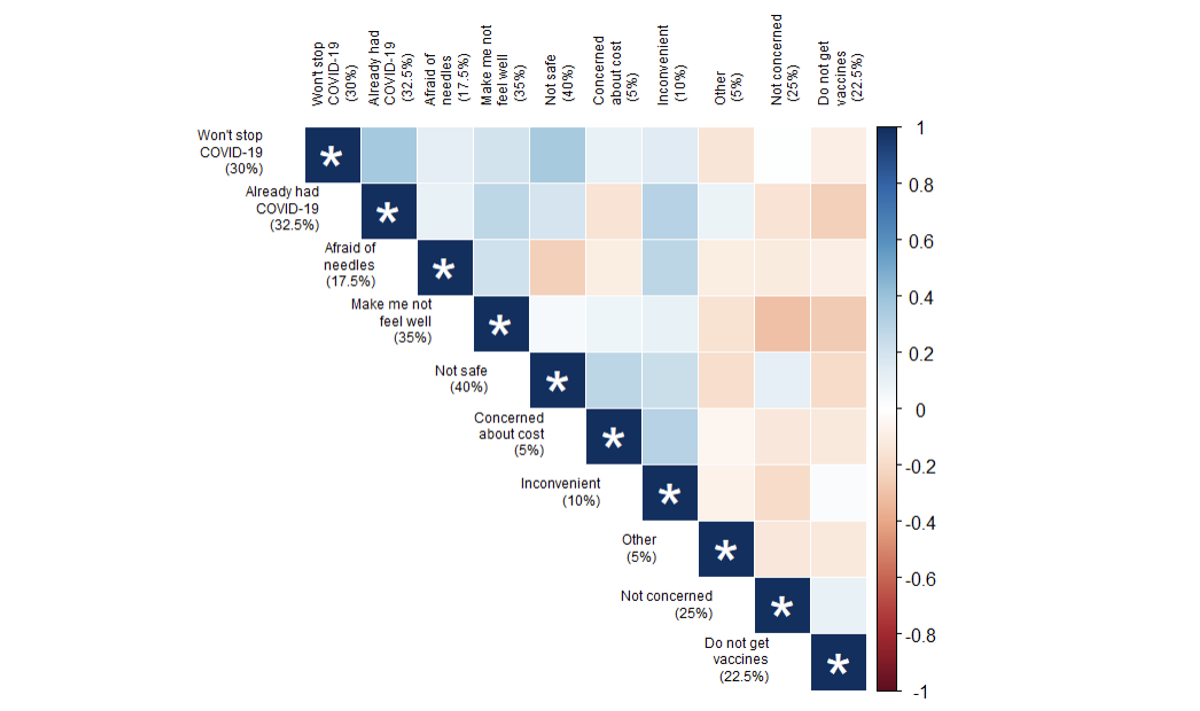
Correlation plot depicting associated and unassociated reasons for expressing hesitancy toward the COVID-19 vaccine among the subsample of autistic adults who were COVID-19 vaccine hesitant in Pennsylvania during 2022. The color of each block represents the strength of the phi coefficient representing a correlation between the intersecting reasons. Phi coefficients with *P*<.05 are symbolized by an asterisk (*). Blue shades capture a tendency for the intersecting reasons to both be reported, contrary to red shades that visualize a tendency for only 1 of the reasons to be reported.

Figures 1-3 report reasons for vaccination and booster acceptance and hesitancy. Among *vaccine and booster accepter* respondents, the most frequent reason reported for accepting both vaccines (139/193, 72%) and boosters (144/193, 74.6%) was the desire to protect others (Figure 1). Most reasons for accepting the vaccine significantly co-occurred, especially among booster doses. For initial vaccination, trust in the vaccine significantly co-occurred with protecting oneself and others. Regarding booster doses, employer, education, or government mandates did not co-occur with any other reason and was the least frequently reported reason for receiving a booster dose besides write-in responses.

The most frequent reason for accepting COVID-19 vaccination among *vaccine accepter, booster hesitant* respondents was that they were encouraged to get a vaccination by someone they trust (17/33, 52%; Figure 2). Almost one-third (10/33, 30%) of this group reported receiving the COVID-19 vaccination due to an employer requirement. The most common reason for COVID-19–vaccinated autistic individuals not to get a booster dose was a belief that their preexisting COVID-19 vaccination status protected them from COVID-19 (15/33, 46%). Over one-quarter (9/33, 27%) reported having COVID-19 and not feeling a booster was necessary or that they were not concerned, and 21.2% (7/33) indicated that that they did not believe the COVID-19 booster would stop COVID-19 occurrence. Almost 1 in 5 (6/33, 18%) reported the vaccine was unsafe or that the COVID-19 vaccination did or might make them feel unwell. Among reasons for booster dose hesitancy, believing that the booster dose was not safe and would not stop the COVID-19 pandemic significantly co-occurred.

The most common reason reported by *vaccine hesitant* respondents was that the vaccine was unsafe (16/40, 40%), followed by believing the vaccine would make them feel unwell (14/40, 35%) or having a previous COVID-19 diagnosis (13/40, 33%; Figure 3). Almost 1 in 3 (12/40, 30%) *vaccine hesitant* autistic adults believed vaccination would not protect against the COVID-19 pandemic. Smaller but substantial groups of autistic adults reported they were not concerned about the COVID-19 pandemic (10/40, 25%), do not get vaccines (9/40, 22%), or are afraid of needles (7/40, 18%).

**Table 1:** Person-level and county-level factors of autistic adults in Pennsylvania sampled in 2022, stratified by self-reported vaccination and booster acceptance or hesitancy.

Factors			Vaccine accepter					Vaccine and booster hesitant (n=40)			*P* value
			Booster accepter (n=193)		Booster hesitant (n=33)						
**Person-level factors**											
	Age (years), mean (SD)		32.3 (9.4)	32.0 (9.0)		32.0 (11.6)			.99		
	**Gender, n (%)^a^**										.03
		Man	138 (72.3)	31 (93.9)		24 (61.5)					
		Woman	46 (24.1)	2 (6.1)		14 (35.9)					
		Write-in response	7 (3.7)	0 (0)		1 (2.6)					
	**Race or ethnicity, n (%)^b^**										.26
		White	137 (79.7)	24 (92.3)		32 (88.9)					
		Non-White	35 (20.3)	2 (7.7)		4 (11.1)					
	**Medical insurance, n (%)^c^**										=.004
		Private	31 (16.8)	4 (12.5)		4 (10.5)					
		Public	92 (50)	7 (21.9)		23 (60.5)					
		Both	61 (33.2)	21 (65.6)		11 (28.9)					
	**Living arrangement, n (%)^d^**										.40
		Family, roommate, and significant other	156 (83.9)	30 (90.9)		36 (90)					
		Independent or other	30 (16.1)	3 (9.1)		4 (10)					
	**Daily routine, n (%)^e^**										.045
		Minor change	27 (14)	4 (12.1)		12 (30.8)					
		Moderate change	36 (18.7)	11 (33.3)		5 (12.8)					
		Major change	32 (16.6)	3 (9.1)		3 (7.7)					
		No change	98 (50.8)	15 (45.5)		19 (48.7)					
	**Exercise routine, n (%)^f^**										.83
		Change	81 (42.2)	15 (45.5)		15 (38.5)					
		No change	111 (57.8)	18 (54.5)		24 (61.5)					
	**Medical care access, n (%)^g^**										.02
		Change	37 (19.2)	0 (0)		7 (17.9)					
		No change	156 (80.8)	33 (100)		32 (82.1)					
	**Behavioral health access, n (%)**										.58
		Change	38 (19.7)	4 (12.1)		7 (17.5)					
		No change	155 (80.3)	29 (87.9)		33 (82.5)					
	**Loneliness, n (%)^h^**										.88
		Increased	59 (30.9)	9 (27.3)		11 (28.2)					
		Same or decreased	132 (69.1)	24 (72.7)		28 (71.8)					
	**Discomfort with others not wearing a mask, n (%)^i^**										.001
		Never or some of the time	140 (73.3)	30 (90.9)		37 (97.4)					
		Most of the time or always	51 (26.7)	3 (9.1)		1 (2.6)					
	**Interacting with too many people impacts my participation in activities, n (%)^j^**										.47
		Never or some of the time	125 (65.8)	25 (75.8)		24 (63.2)					
		Most of the time or always	65 (34.2)	8 (24.2)		14 (36.8)					
	**Previous COVID-19 diagnosis (1 or more), n (%)**										.001
		Yes	39 (20.2)	11 (33.3)		19 (47.5)					
		No	154 (79.8)	22 (66.7)		21 (52.5)					
	**Know someone with adverse outcomes due to COVID-19, n (%)**										.95
		Yes	69 (35.8)	11 (33.3)		12 (30)					
		No	124 (64.2)	22 (66.7)		28 (70)					
	**Changes in interactions with family or friends, n (%)^k^**										.21
		Yes	58 (30.2)	7 (21.2)		7 (17.9)					
		No	134 (69.8)	26 (78.8)		32 (82.1)					
	**COVID-19 impact on community participation in the last 30 days, n (%)**										.22
		Stayed the same	74 (38.3)	14 (42.4)		19 (47.5)					
		Increased	13 (6.7)	2 (6.1)		6 (15)					
		Decreased	106 (54.9)	17 (51.5)		15 (37.5)					
	**Stress related to the COVID-19 pandemic, n (%)**										.08
		No stress	40 (20.7)	6 (18.2)		14 (35)					
		Mild stress	76 (39.4)	16 (48.5)		12 (30)					
		Moderate stress	52 (26.9)	3 (9.1)		8 (20)					
		Major stress	25 (13)	8 (24.2)		6 (15)					
	**Sources used to get COVID-19 information (select all), n (%)**										
		Social media	88 (45.6)	15 (45.5)		16 (40)			.81		
		Cable news	50 (25.9)	8 (24.2)		8 (20)			.73		
		Local news	95 (49.2)	10 (30.3)		15 (37.5)			.08		
		National elected officials	32 (16.6)	3 (9.1)		3 (7.5)			.22		
		Local elected officials	21 (10.9)	1 (3)		3 (7.5)			.33		
		I did my own research	56 (29)	11 (33.3)		12 (30)			.88		
		I did not try to access information about COVID-19	24 (12.4)	9 (27.3)		15 (37.5)			.001		
		Other	47 (24.4)	6 (18.2)		5 (12.5)			.22		
**County-level factors, mean (SD)**											
	2020 average total population		614,000 (512)	441,000 (411)		384,000 (336)			.02		
	2020 Biden vote (%)		52.5 (16.2)	45.6 (15)		42.8 (14)			.001		

^a^Vaccine and booster accepter: n=191; vaccine and booster hesitant: n=39.

^b^Vaccine and booster accepter: n=172; vaccine accepter and booster hesitant: n=26; vaccine and booster hesitant: n=36.

^c^Vaccine and booster accepter: n=184; vaccine accepter and booster hesitant: n=32; vaccine and booster hesitant: n=38.

^d^Vaccine and booster accepter: n=186.

^e^Vaccine and booster hesitant: n=39.

^f^Vaccine and booster accepter: n=192; vaccine and booster hesitant: n=39.

^g^Vaccine and booster hesitant: n=39.

^h^Vaccine and booster accepter: n=191; vaccine and booster hesitant: n=39.

^i^Vaccine and booster accepter: n=191; vaccine and booster hesitant: n=38.

^j^Vaccine and booster accepter: n=190; vaccine and booster hesitant: n=38.

^k^Vaccine and booster accepter: n=192; vaccine and booster hesitant: n=39.

## Discussion

### Principal Findings

Study findings reveal new information about COVID-19 vaccination and booster perspectives and decisions among the growing group of autistic adults. Although participants do not perfectly align with the characteristics of Pennsylvania, limiting comparisons, autistic adults in our sample had higher rates of intended initial vaccination than the initial vaccination rate among the general population in Pennsylvania (87.6% vs 71.8%) [36,37]. Booster acceptance among our sample also more than doubled the booster dose rate (72.6% vs 30.2%) [36,37]. Mirroring population trends and previous research [7], population density and political makeup strongly correlated with vaccine acceptance and hesitancy. COVID-19 vaccines have become immensely politicized in the United States [38] as political party affiliation is increasingly associated with vaccination status [12], and our results highlight that autistic adults are not immune to partisan views on vaccines and may even be deeply swayed by them. Those accepting of initial vaccination and boosters were found to have different motivations than those accepting of the former but hesitant of the latter. *Vaccine and booster accepter* respondents were guided by their desire to protect others. *Vaccine accepter, booster hesitant* participants, however, were most often following the guidance of someone they trusted for their initial COVID-19 vaccine. *Vaccine hesitant* individuals, similar to findings in the general population [39,40], most frequently cited their belief that the vaccines were unsafe, which was closely followed by the fear that the vaccine would make them feel unwell.

Importantly, our data rely on self-report, extending research that has focused on vaccination preferences of autistic children [41] and disabilities generally [24,42,43]. Most research on autistic adults relies upon caregiver proxies, which lacks the full input or perspective of autistic adults directly [44]. Prioritizing self-reported data ensures that participants take an active role and report their experiences, a crucial need within vaccine research. Hearing directly from autistic adults about their COVID-19 vaccination beliefs and preferences, and extending this work across public health, is critical for health care providers and for public health messaging to be understood, embraced, and support the decision-making processes.

The desire to be informed about the COVID-19 pandemic significantly impacted vaccination preferences, with the *vaccine hesitant* group least likely to access any COVID-19 information. These individuals may be less likely to encounter credible health sources, and COVID-19 misinformation may have filled this information vacuum, driving hesitancy. For instance, the most commonly endorsed reason for vaccine hesitancy was that it was not safe. It is critical that public health messaging address falsehoods absorbed by the public, particularly given the frequency of misinformation exposure among those not actively seeking information. For maximum transparency, messaging should balance the fact that side effects are possible yet are a considerably better outcome than contracting COVID-19. Traditional information dissemination channels cannot counter the myriad of misinformation within the COVID-19 discourse. Social media, however, can be a powerful tool, and our results show that social media use did not differ by vaccination preference. Using social media for public health messaging necessitates a coherent messaging strategy to ensure impact [45], with an approach tailored to those with diverse processing needs, and who have differing experiences and perspectives.

Conversely, the *vaccine and booster accepter* group expressed an altruistic reason as the most often cited reason for receiving the vaccine—protecting others. This was followed by trust in the vaccine, with both reasons coming before concerns of protecting themselves from serious illness and infection. Meanwhile, *vaccine accepter, booster hesitant* individuals appeared more externally motivated for accepting initial vaccination, as nearly half noted that they were encouraged by someone they trust to receive the vaccination. This group was also nearly half as likely to endorse protecting others as a motivating factor compared to the *vaccine and booster accepter* group, which likely influenced their later hesitancy to booster doses. The same could be true for the greater influence employer or education setting mandates had, as these policies have relaxed or been eliminated.

Still, with low booster rates across the country [21], it is important to identify the motivations among those who receive or reject boosters, especially from vulnerable populations. *Vaccine and booster accepter* respondents had markedly similar beliefs about initial vaccination and booster doses, suggesting that their vaccination views remained consistent as the pandemic evolved; protecting others remained the top reason for acceptance. Extant research shows that among young adults in the general population, messaging that focuses on protecting other’s health through vaccination can be an effective motivator [46]. Those findings align with these study results and are an important takeaway for public health practitioners. Booster hesitancy was rooted in the belief that protection from the first vaccine was retained, followed by a lack of concern for the COVID-19 pandemic and a previous COVID-19 diagnosis. It is clear that different strategies are needed to reach this group.

Although our overall sample skewed toward men (193/266, 72.6% vs 62/266, 23.3%), a greater proportion of autistic women were *vaccine hesitant* than men (14/62, 23% vs 24/193, 12.4%). This counters the general population in Pennsylvania, where recent estimates indicate 61.5% of men are vaccinated compared to 69% of women [23]. While autism is more commonly diagnosed in boys, the experiences that autistic girls and women have with the health care system may contribute to increased hesitancy. Emerging research suggests that girls are underdiagnosed due to different presentations [47] and a health care system that is not attuned to their experiences. Autistic adults report worse health care quality and health than the general population [48], and autistic women experience increased barriers to services than autistic men [49]. This multiplicative disproportionality may increase medical mistrust among autistic women. Taken together, these findings serve as an additional reminder that current health infrastructure is ill-equipped to support autistic women and public health messaging should consider how gender shapes the experiences and perspectives of autistic people.

Health insurance coverage was significantly associated with differing vaccination perspectives as autistic adults enrolled in private insurance were most likely to be accepting, while autistic adults who were vaccine and booster hesitant or booster hesitant were often enrolled in public insurance (Medicaid). Considering low rates of employment among autistic adults and associated reduced access to employer-sponsored private insurance, Medicaid is a critical safety net for autistic people across the lifespan [50]. Our results suggest that the Medicaid system would benefit from more proactively promoting vaccine acceptance to enrollees via increasing access to COVID-19 vaccines and dissemination of plain-language information on the importance of being vaccinated against COVID-19. Pennsylvania is uniquely situated for this role, as the state has 2 of the first adult-only (age 21 years and older) autism-specific Medicaid programs in the United States (the Adult Autism Waiver and the Adult Community Autism Program), as well as other Medicaid waivers that enroll autistic adults and do not require a co-occurring intellectual disability diagnosis. However, there are waiting lists for all of these programs, totaling thousands of autistic adults [51], indicating that more outreach is needed to non-waiver enrolled Medicaid beneficiaries. Connections to health insurance indicate that there are likely to be interactions with health or behavioral health care providers, services, and supports that may serve as conduits for health promotion opportunities to support vaccination or to provide information to address fears, including needle anxiety. For this Medicaid-reliant population, the ending of the COVID-19 public health emergency is impactful. The termination of the public health emergency designation for COVID-19 includes the expiration of coverage for many options for COVID-19 testing, treatment, and vaccinations. Disaster relief state plan amendments and waiver modifications in the Medicaid system aimed at increasing eligibility and enrollment flexibility ended [52].

Half of autistic adults reported no daily routine changes during the COVID-19 pandemic and disruptions were more often reported by the *vaccine and booster accepter* group. With less impact on day-to-day living and beliefs that the vaccine is unsafe, will not stop the COVID-19 pandemic, or will make recipients feel unwell, it is plausible the *vaccine hesitant* group did not see enough tangible benefit to outweigh vaccine-related concerns, including the incentive that getting back to prepandemic routines may have provided. Meanwhile, the *vaccine accepter, booster hesitant* group’s booster hesitancy was most often due to a belief that initial vaccines and prior COVID-19 diagnoses provided protection. These findings suggest that, among autistic adults, different messaging strategies are needed for those who reject all vaccines, versus those who have yet to receive a booster dose. Evidence-based strategies include multimodal information delivery; plain, accessible language; and using a variety of dissemination channels [53]. However, the best blend of these strategies for maximum impact on autistic adults remains a research priority.

### Limitations

Although this study has significant strengths, including collection of data directly from autistic adults, includes limitations. The results of this study are not population-level data for autistic adults, and it is possible that individuals who did not respond or were not recruited to participate, have differing vaccination perspectives than our sample. Our sample would also benefit from additional diversity. The COVID-19 pandemic has further illuminated racial and ethnic health disparities, and these experiences impact how adults perceive vaccines. It may also have impacted our survey response rate. A greater proportion of non-White respondents, however, reported being or intending to become fully vaccinated, but our sample lacked sufficient power to detect statistical significance. Although only 15.4% (41/266) of our sample were individuals identifying as racial minorities, 85% (35/41) of these respondents were fully vaccinated and boosted, compared to 70.2% (158/225) of the remaining respondents. Future research must prioritize oversampling autistic adults identifying as racial minorities, as the intersection of disability and race can yield additive marginalization. A more thorough exploration of age differences is another important direction for future research, given the impact the COVID-19 pandemic has on older adults. Effective messaging strategies for this group may differ from younger peers.

### Conclusions

Our study results have significant public health implications. The service system landscape is currently providing insufficient care to autistic adults [54] and remains unprepared for the significant rise in this population. Maximum vaccine protection is important to ensure a healthier autistic adult population that can aid a strained service system’s ability to offer adequate care, as autistic adults, like the larger disability community, are at elevated risks for contracting COVID-19 and experiencing adverse outcomes [3]. Although no longer declared a public health emergency, as of June 2024, US health officials are still urging caution and planning for the potential, for a rise of cases as new variants emerge [55,56]. With the COVID-19 pandemic remaining prevalent, the collision of the virus and service delivery for autistic adults poses serious public health concerns. Still, across the spectrum, the autism community is diverse, with various opinions and views about health care. Decision-making agency among autistic adults must be prioritized, which highlights the importance of engaging with autistic adults directly.

To support the health autonomy of autistic adults, COVID-19 information, especially regarding vaccines, must be accessible. Adults fully hesitant of COVID-19 vaccines were significantly more likely to not access COVID-19 information and to report that vaccination hesitancy was due to the (inaccurate) belief that vaccines are unsafe. Misinformation, especially around the COVID-19 pandemic, is pervasive; it is likely that individuals are exposed to it despite not actively seeking COVID-19 information. Public health officials need to be cognizant and proactively responsive to passive absorption of misinformation and adopt messaging strategies that target individuals who are not actively seeking information. Evidence also suggests that efforts to target COVID-19 misinformation and address emerging conspiracy theories may be more effective at increasing vaccine and booster acceptance than COVID-19 vaccination or booster mandates [57]. Timely, trustworthy, and transparent messaging can help restore credibility and promote health decision-making rooted in evidence and fact.

## Abbreviations

ICD: International Classification of Diseases
KFF: Kaiser Family Foundation
PANA: Pennsylvania Autism Needs Assessment

## Data Availability

The data sets generated and analyzed during this study are not publicly available out of respect for our participants but are available from the corresponding author on reasonable request and with the required and appropriate confidentiality and safety restrictions.
